# Serum biomarker profile orchestrating the seroconversion status of patients with autoimmune diseases upon planned primary 17DD Yellow fever vaccination

**DOI:** 10.1038/s41598-021-89770-8

**Published:** 2021-05-17

**Authors:** Ismael Artur da Costa-Rocha, Ketty Lysie Libardi Lira Machado, Ana Carolina Campi-Azevedo, Andréa Teixeira-Carvalho, Vanessa Peruhype-Magalhães, Sheila Maria Barbosa de Lima, Emily Hime Miranda, Gisela Freitas Trindade, Thays Zanon Casagrande, Samira Tatiyama Miyamoto, Sávio Carvalho Deotti, Rafaela Villa Real Barbosa, Priscila Costa Martins Rocha, Erica Vieira Serrano, Valquiria Garcia Dinis, Sônia Alves Gouvêa, Maria Bernadete Renoldi de Oliveira Gavi, Lidia Balarini da Silva, Ruben Horst Duque, Ana Paula Espíndula Gianordoli, Maria de Fatima Bissoli, Maria da Penha Gomes Gouvea, Lauro Ferreira da Silva Pinto-Neto, Ana Paula Neves Burian, Francieli Fontana Sutile Tardetti Fantinato, Gecilmara Salviato Pileggi, Licia Maria Henrique da Mota, Valéria Valim, Olindo Assis Martins-Filho

**Affiliations:** 1 Grupo Integrado de Pesquisas Em Biomarcadores, Instituto René Rachou, Fundação Oswaldo Cruz (FIOCRUZ-Minas), Avenida Augusto de Lima 1715, Barro Preto, Belo Horizonte, MG 30190-002 Brazil; 2 Hospital Universitário Cassiano Antônio de Moraes, Universidade Federal Do Espírito Santo (UFES), Av. Mal. Campos, 1355, Santos Dumont, Vitória, ES 29041-295 Brazil; 3 Instituto de Tecnologia Em Imunobiológicos (Bio-Manguinhos), Fundação Oswaldo Cruz (FIOCRUZ), Rio de Janeiro, RJ Brazil; 4 Escola de Ciências da Saúde da Santa Casa de Misericórdia, Vitória, ES Brazil; 5 Centro de Referências Para Imunobiológicos Especiais (CRIE) da Secretaria de Saúde Do Estado Do Espírito Santo, Vitória, ES Brazil; 6 Departamento de Vigilância das Doenças Transmissíveis, Secretaria de Vigilância Em Saúde, Ministério da Saúde, Brasília, DF Brazil; 7 Faculdade de Ciências da Saúde de Barretos – FACISB, Barretos, SP Brazil; 8 Programa de Pós Graduação Em Ciências Médicas, Faculdade de Medicina, Serviço de Reumatologia Do Hospital Universitário de Brasília, Universidade de Brasília, Brasília, DF Brazil

**Keywords:** Biomarkers, Immunology, Autoimmunity, Chemokines, Cytokines, Vaccines

## Abstract

The present study aimed to investigate whether the serum biomarkers of immune response orchestrate the seroconversion status in patients with autoimmune diseases (AID) upon planned primary 17DD-YF vaccination. For this purpose a total of 161 individuals were enrolled in a prospective study, including patients with Rheumatoid Arthritis (RA = 38), Spondyloarthritis (SpA = 51), Systemic Lupus Erythematosus (SLE = 21) and Sjögren’s Syndrome (SS = 30) along with a group of healthy controls (HC = 21). Analysis of plaque reduction neutralization test (PRNT) titers and seropositivity rates along with the 17DD-YF viremia and serum biomarkers were carried out at distinct time points (D0/D3–4/D5–6/D7/D14–28). The results demonstrated an overall lower PRNT titer and seropositivity rate (170 vs. 448; 77 vs. 95%) in AID as compared to HC, especially in SpA and SLE subgroups. No significant differences were observed in the viremia levels amongst groups. In general, a more prominent serum biomarker response was observed in AID as compared to HC, throughout the timeline kinetics. Remarkably, AID/PRNT(−) exhibited higher levels of several biomarkers at baseline as compared to AID/PRNT+. Moreover, while AID/PRNT(+) exhibited earlier increase in serum biomarkers at D3–4/D5–6, the AID/PRNT(−) displayed higher response at later time points (D7/D14–D28). Of note, a synchronic increase of IFN-γ at the peak of viremia (D5–6) was observed in HC and AID/PRNT(+) groups, whereas a later asynchronous IFN-γ response was reported for AID/PRNT(−) at D7. The biomarker profile tends to deflate at post-vaccination timeline, highlighting a putative immunomodulatory effect of live attenuated 17DD-YF vaccine in AID/PRNT(+), but not in AID/PRNT(−). Altogether these data suggested that inflammatory status prior vaccination, low IFN-γ at viremia peak and the occurrence of asynchronous biomarker storm after 17DD-YF vaccination may orchestrate the lack of neutralizing antibody response γ.

## Introduction

Yellow fever (YF) is a vector-borne disease caused by RNA arboviruses of the family Flaviviridae, transmitted by *Haemagogus*, *Sabethes* and *Aedes* arthropods. The disease is endemic in several tropical areas of South America and Africa, presenting a broad clinical spectrum with mortality rates approach 50% in patients with severe disease^1,2^. The number of YF cases is estimated at 80,000–200,000 per year, with approximately 30,000–60,000 deaths worldwide^1,2^. The eradication of YF is unfeasible due to the sylvatic reservoir system and the treatment with approved antiviral is unavailable^3,4^. In this sense, the large-scale vaccination coverage is the only effective measure to control the disease spread and reemergence of epidemic outbreaks^5^. The live-attenuated YF vaccine was developed in 1937 and two substrains (17D and 17DD) are currently available worldwide^6^. The 17D and 17DD-YF vaccines are safe and highly immunogenic, inducing protective immunity in approximately 95% of healthy adults upon primary vaccination^7^.

In general, it has been proposed that most vaccines are immunogenic for AID patients. However, some vaccines are shown to be less effective in AID and/or in patients receiving immunomodulatory therapy when compared to healthy control subjects^8^. In fact, it has been reported that YF vaccination induces a suboptimal immunologic response in patients with autoimmune diseases^9–11^. Conversely, other studies have pointed out that the immunogenicity of YF vaccine reported for AID patients are similar to that observed in healthy controls^12^.

Aiming at providing a safe planned YF vaccination for patients with chronic immune-mediated inflammatory diseases, the Brazilian Society of Rheumatology, Dermatology, Bowel Inflammatory Disease have recently released a guideline defining the individual risk/benefit and establishing personalized recommendations^13^. It has been posted that in situations of risk, when the YF vaccination is indicated, a minimum withdrawal period for immunosuppressant or biological therapy should be considered, according to the immunosuppression degree and upon advice of a rheumatologist^13^.

Based on this guideline, our group has previously developed a prospective non-interventional study to assess the safety and immunogenicity of planned 17DD-YF primary vaccination in AID patients^14^. Consistent seroconversion rates (78%) were observed in AID patients, supporting the safety and immunogenicity of planned 17DD-YF primary vaccination for AID patients. However, AID patients presented a lower seropositivity rate as compared to healthy controls (96%), especially those patients with Spondyloarthritis (SpA = 73%) and Systemic Lupus Erythematosus (SLE = 73%). Moreover, an overall late seroconversion profile at Day28 was observed for AID patients while most healthy controls (75%) already displayed seropositivity at Day14 along the timeline kinetics of neutralizing antibodies. Similar viremia profiles did not substantiate the differences observed in the seroconversion rates. The analysis of additional immunological parameters may bring novels insights to elucidate the differential seroconversion profiles observed amongst AID patients.

The present study aimed to investigate whether the serum biomarkers of immune response orchestrate the seroconversion status in AID patients, leading to positive or negative plaque reduction neutralization test (PRNT), depending on the type of AID. We hypothesize that serum soluble mediator microenvironment at baseline and the fold changes upon planned 17DD-YF primary vaccination are closely related to the seroconversion outcome in the AID patients. Our findings support that the baseline biomarker status prior vaccination as well as the synchronic/asynchronous rhythm of soluble mediators nearby the viremia peak orchestrate the seroconversion status of AID patients upon planned primary 17DD-YF vaccination.

## Results

### Seropositivity rates and neutralizing antibodies levels in patients with autoimmune disease upon planned primary 17DD-YF vaccination

The quantification of YF-specific neutralizing antibody was carried out using the standard protocol of plaque-reduction neutralization test (PRNT) and the results are shown in the Table 1. Data analysis demonstrated that the seropositivity rate was significant lower in AID patients (77%) as compared to HC (95%). Subsequent categorization of AID patients according to the type of autoimmune disease further demonstrated that SpA (73%) and SLE (71%) presented lower seropositivity rates, while RA (87%) and SS (77%) displayed similar seropositivity rates as compared to HC. The analysis of neutralizing antibodies levels (GeoMean) corroborate the seropositivity rates, showing lower PRNT titers in AID (170), SpA (112) and SLE(133), but similar levels in RA (291) and SS (209) as compared to HC (448) (Table 1).

**Table 1:** Seropositivity Rates and Neutralizing Antibodies Levels in Patients with Autoimmune Disease upon Planed Primary 17DD-YF Vaccination.

Study groups	Seropositivity rates % (n)	GeoMean (95%CI)
Health controls (HC)	95% (20/21)	448 (285–705)
Autoimmune diseases (AID)	77% (108/140)*	170 (133–219)^#^
Rheumatoid Arthritis (RA)	87% (33/38)	291 (194–436)
Spondyloarthritis (SpA)	73% (37/51)*	112 (73–170)^#^
Systemic Lupus Erythematosus (SLE)	71% (15/21)*	133 (55–321)^#^
Sjögren’s Syndrome (SS)	77% (23/30)	209 (115–378)

Data are reported as the proportion of seropositivity upon planed primary 17DD-YF vaccination and geometric mean (GeoMean) of plaque-reduction neutralizing test (PRNT). * Significant difference at *p* < 0.05 by χ^2^ test. ^#^ Significant difference at *p* < 0.05 by Kruskal–Wallis analysis followed by Dunn’s post test.

### Viremia levels in patients with autoimmune disease upon planned primary 17DD-YF vaccination

The viremia level quantification (YF Viral RNAnemia) was performed by qRT-PCR assay and the data expressed as mean copies/mL at peak of viremia are presented in the Table 2. The results demonstrated that all groups exhibited the peak of viremia around Day5–6 after vaccination. The analysis of viremia levels at peak did not shown significant differences amongst AID subgroups as compared to HC (Table 2).

**Table 2:** Viremia Levels in Patients with Autoimmune Disease upon Planed Primary 17DD-YF Vaccination.

Study groups	Viremia peak (day after vaccination)	Viremia level at peak (mean copies/mL)*	*p* Value
Health controls (HC) (n = 13)	Day 5–6	4.8 ± 3.8 × 10^3^	> 0.9999
Autoimmune diseases (AID) (n = 86)	Day 5–6	5.5 ± 2.1 × 10^3^	> 0.9999
AID/PRNT(−) (n = 15)	Day 5–6	0.8 ± 0.6 × 10^3^	> 0.9999
AID/PRNT(+) (n = 71)	Day 5–6	6.6 ± 2.5 × 10^3^	> 0.9999
Rheumatoid Arthritis (RA) (n = 24)	Day 5–6	1.6 ± 0.7 × 10^3^	> 0.9999
Spondyloarthritis (SpA) (n = 29)	Day 5–6	6.1 ± 3.2 × 10^3^	> 0.9999
Systemic Lupus Erythematosus (SLE) (n = 11)	Day 5–6	2.7 ± 2.6 × 10^3^	> 0.9999
Sjögren’s Syndrome (SS) (n = 18)	Day 5–6	12.9 ± 8.4 × 10^3^	> 0.9999

* Data are reported as mean copies of 17DD-YF RNAnemia at peak ± standard error. No significant difference by no-parametric Kruskal–Wallis analysis (*p* = 0.5056) followed by Dunn’s post-test for sequential pairwise comparisons and a threshold *p* value of < 0.05 was considered for statistical significance.

### Timeline kinetics of serum biomarkers in patients with autoimmune disease following planned primary 17DD-YF vaccination

**Figure 1:**
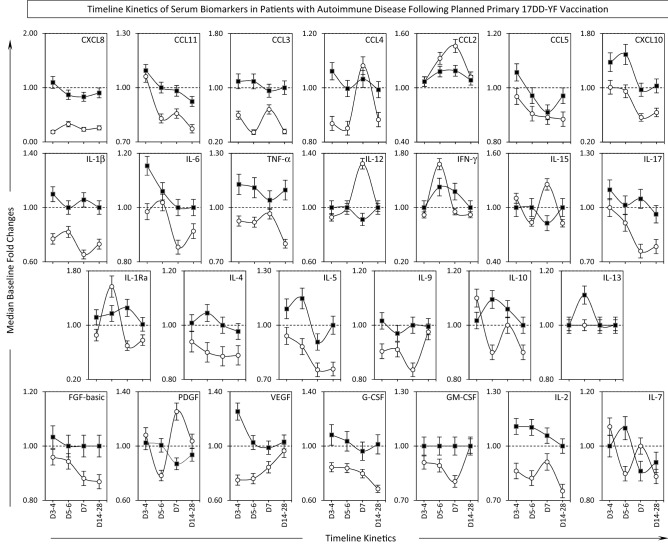
Timeline Kinetics of Serum Biomarkers in Patients with Autoimmune Disease Following Planned Primary 17DD-YF Vaccination. Panoramic timeline kinetics of serum chemokines (CXCL8, CCL11, CCL3, CCL4, CCL2, CCL5, CXCL10), pro-inflammatory cytokines (IL-1β, IL-6, TNF-α, IL-12, IFN-γ, IL-15, IL-17), regulatory cytokines (IL-1Ra, IL-4, IL-5, IL-9, IL-10, IL-13), and growth factors (FGF-basic, PDGF, VEGF, G-CSF, GM-CSF, IL-2 and IL-7) in serum samples from Autoimmune Disease patients (AID, black filled square, n = 140) and Healthy Controls (HC, white filled circle, n = 21). Measurements were carried out using the Luminex platform, according to manufacturer’s instructions as provided in Material and Methods. The results are expressed as median baseline fold changes ± variation at each time point along the kinetic follow-up (D3–4, D5–6, D7 and D14–28) according to the paired sample collected at D0. Data analysis was performed considering the baseline fold value = 1.0 as the reference for decrease (< 1.0) or increase (> 1.0) in biomarker levels along the kinetic timeline.

The quantification of serum chemokines, pro-inflammatory cytokines, regulatory cytokines and growth factors was carried out by high-performance microbeads array at baseline (Day0) and at four consecutive scheduled time points, including: Day3–4, Day5–6, Day7 and Day14–D28 after vaccination. The results are presented as median baseline fold changes in the Fig. 1. Data analysis demonstrated that 17DD-YF vaccine triggered a more prominent serum biomarker response in AID as compared to HC, throughout the timeline kinetics. In general, prominent increase in baseline fold changes observed in AID patients occurred at Day3–4 (CXCL8, CCL11, CCL3, CCL4, CCL5, IL-1β, IL-6, TNF-α, IL-17, IL-9, FGF-basic, VEGF, G-CSF, IL-2), at Day5–6 (CXCL10, IL-4, IL-5, IL-10, IL-13, IL-7) or at Day7 (IL1Ra). In a few situations along the timeline kinetics AID presented lower baseline fold changes as compared to HC, as observed at Day3–4 (IL-15, IL-10, PDGF, IL-7), at Day5–6 (CCL2, IFN-γ, IL1Ra) and at Day7 (CCL4, CCL2, IL-12, IL-15, PDGF, IL-7). Overall, the baseline fold changes were higher in AID at Day14–28 as compared to HC. Of note, differential kinetic profile of IFN-γ and IL-10 was observed for HC and AID. While HC showed high IFN-γ but low IL-10 at Day5–6, lower increase of IFN-γ concomitant with up-regulation of IL-10 was observed to AID at Day5–6 (Fig. 1). These differences in baseline fold changes profiles of serum biomarkers detected along the timeline kinetic and particularly at the peak of viremia (Day5–6) may explain the distinct seroconversion profile observed in AID as compared to HC.

### Timeline kinetics of serum biomarkers in patients with autoimmune disease according to the neutralizing antibody status after planned primary 17DD-YF vaccination

**Figure 2:**
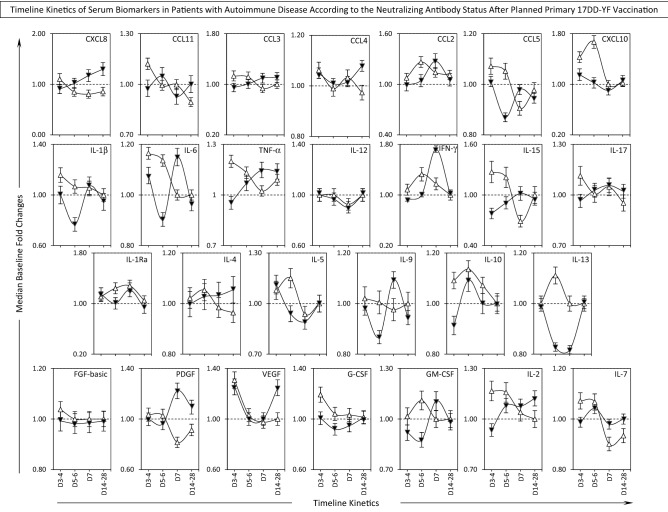
Timeline Kinetics of Serum Biomarkers in Patients with Autoimmune Disease According to the Neutralizing Antibody Status After Planned Primary 17DD-YF Vaccination. Panoramic timeline kinetics of serum chemokines (CXCL8, CCL11, CCL3, CCL4, CCL2, CCL5, CXCL10), pro-inflammatory cytokines (IL-1β, IL-6, TNF-α, IL-12, IFN-γ, IL-15, IL-17), regulatory cytokines (IL-1Ra, IL-4, IL-5, IL-9, IL-10, IL-13), and growth factors (FGF-basic, PDGF, VEGF, G-CSF, GM-CSF, IL-2 and IL-7) in serum samples from Autoimmune Disease patients, categorized according to the PRNT status after primary 17DD-YF vaccination: AID/PRNT(−) (black filled inverted triangle, n = 32) and AID/PRNT(+) (white filled inverted triangle, n = 108). Measurements were carried out using the Luminex platform, according to manufacturer’s instructions as provided in Material and Methods. The results are expressed as median baseline fold changes ± variation at each time point along the kinetic follow-up (D3–4, D5–6, D7 and D14–28) according to the paired sample collected at D0. Data analysis was performed considering the baseline fold value = 1.0 as the reference for decrease (< 1.0) or increase (> 1.0) in biomarker levels along the kinetic timeline.

The timeline kinetics of changes in serum biomarkers were further evaluated in AID patients categorized according to the PRNT status after primary 17DD-YF vaccination referred as AID/PRNT(−) (n = 32) and AID/PRNT(+) (n = 108) and the results showed in the Fig. 2. Data analysis demonstrated that, in general, a more prominent serum biomarker response was observed in AID/PRNT(+) as compared to AID/PRNT(−), throughout the timeline kinetics. Prominent early increase in baseline fold changes observed in AID/PRNT(+) patients occurred at Day3–4 (CXCL8, CCL11, CCL5, IL-1β, IL-6, TNF-α, IL-15, IL-17, FGF-basic, G-CSF, IL-2, IL-7) and at Day5–6 (CCL2, CXC10, IFN-γ, IL-5, IL-10, IL-13, GM-CSF) as compared to Day7 (IL1Ra). Conversely, at late time points along the timeline kinetics, the AID/PRNT(+) presented lower baseline fold changes as compared to AID/PRNT(−), as observed at Day7 (CXCL8, CCL3, CCL2, CCL5, IL-6, TNF-α, IFN-γ, IL-15, IL-4, IL-9, PDGF, CM-CSF, IL-2, IL-7) as compared to Day5–6 (CXCL8, CCL11). Moreover, the AID/PRNT(−) exhibited increased levels of serum biomarkers late at Day14–28 as observed for (CXCL8, CCL11, CCL3, CCL4, TNF-α, IL-17, IL-4, PDGF, VEGF, IL-2, IL-7). Of note, a synchronic pattern of high IFN-γ levels at the day of peak viremia (Day5–6) was observed in AID/PRNT(+) groups, whereas an asynchronous IFN-γ response was reported for AID/PRNT(−) later at Day7. However, both subgroups exhibited peak of IL-10 concomitant with the viremia peak at Day5–6 (Fig. 2).

### Baseline profile of serum biomarkers in patients with autoimmune disease according to the neutralizing antibody status after planned primary 17DD-YF vaccination

**Figure 3:**
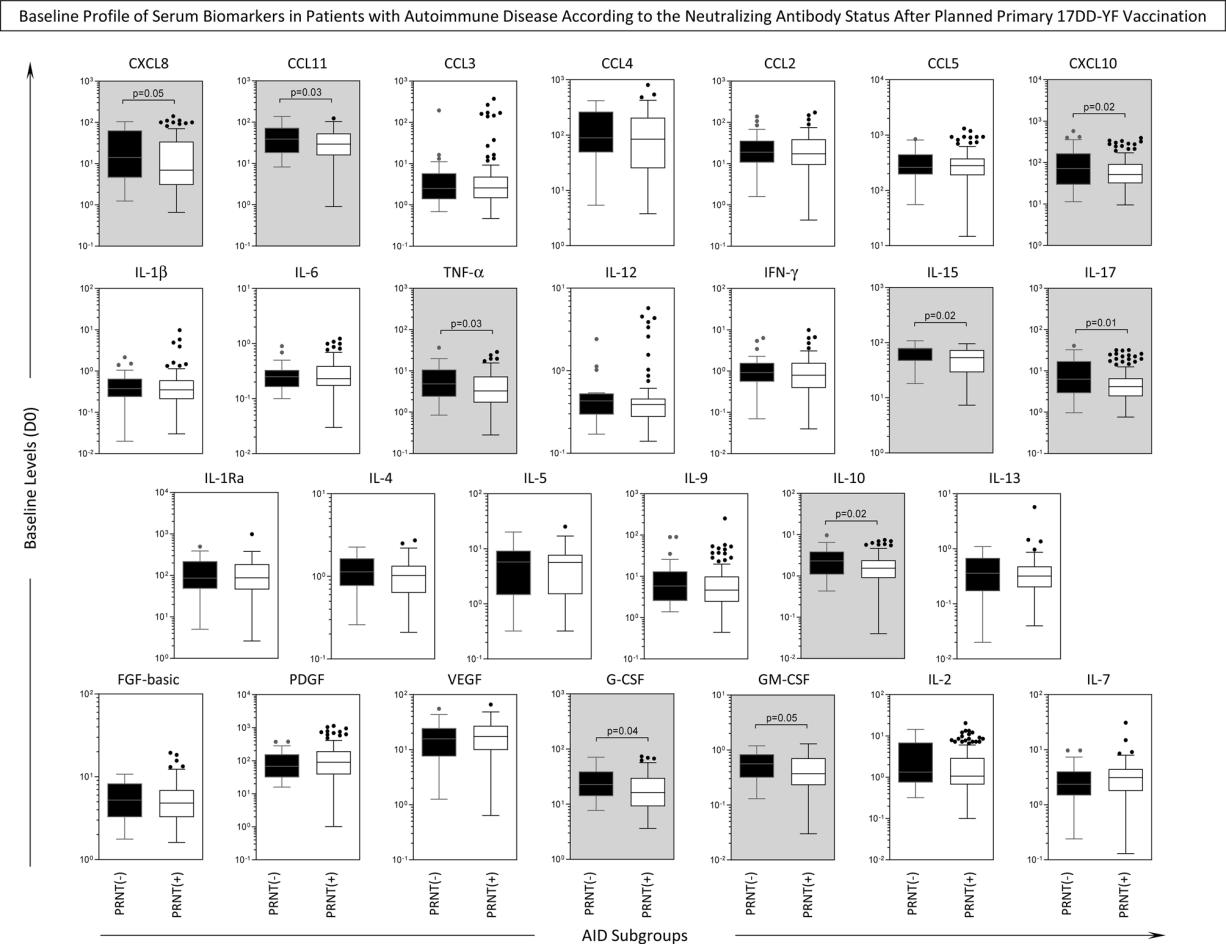
Baseline Profile of Serum Biomarkers in Patients with Autoimmune Disease According to the Neutralizing Antibody Status After Planned Primary 17DD-YF Vaccination. Baseline levels of serum chemokines (CXCL8, CCL11, CCL3, CCL4, CCL2, CCL5, CXCL10), pro-inflammatory cytokines (IL-1β, IL-6, TNF-α, IL-12, IFN-γ, IL-15, IL-17), regulatory cytokines (IL-1Ra, IL-4, IL-5, IL-9, IL-10, IL-13), and growth factors (FGF-basic, PDGF, VEGF, G-CSF, GM-CSF, IL-2 and IL-7) in serum samples from Autoimmune Disease patients, categorized according to the PRNT status after primary 17DD-YF vaccination: AID/PRNT(−) (black filled square, n = 32) and AID/PRNT(+) (white filled square, n = 108). Measurements were carried out using the Luminex platform, according to manufacturer’s instructions as provided in Material and Methods. The results are expressed as median levels (pg/mL) at baseline (D0) on boxplot charts. Comparative analysis between AID/PRNT(−) and AID/PRNT(+) was carried out by Mann–Whitney test and significant differences at *p* < 0.05 underscored by connecting lines and highlight with gray background. The exact *p* values are provided in the figure.

Aiming at investigating whether the Day0 baseline profile of serum biomarker was associated with the seroconversion status achieved after planned primary 17DD-YF vaccination, the AID patients were categorized according to the PRNT status after primary 17DD-YF vaccination as AID/PRNT(−) and AID/PRNT(+) and the biomarker levels prior planned primary 17DD-YF vaccination compared between AID subgroups. The results are presented in the Fig. 3. Data analysis demonstrated that AID/PRNT(−) exhibited higher levels of several biomarkers at Day0 baseline (CXCL8, CCL11, CXCL10, TNF-α, IL-15, IL-17, IL-10, G-CSF, GM-CSF) as compared to AID/PRNT(+), suggesting that an inflammatory status prior vaccination may orchestrated the lack of antibody response (Fig. 3).

### Serum biomarker signature in patients with autoimmune disease after planned primary 17DD-YF vaccination

**Figure 4:**
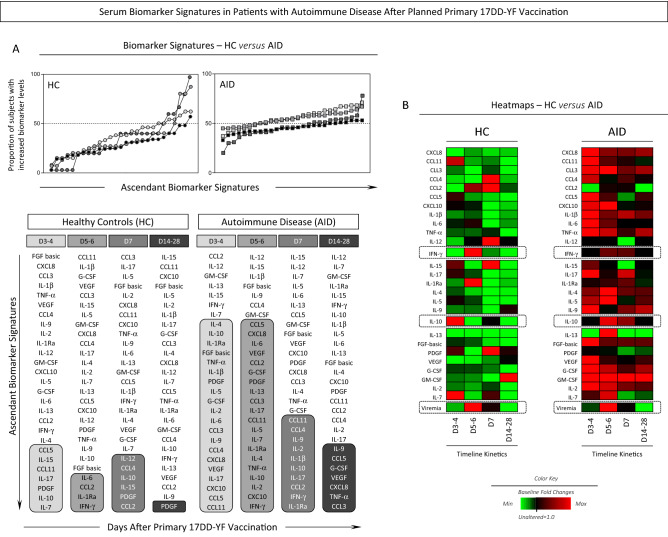
Serum Biomarker Signatures in Patients with Autoimmune Disease After Planned Primary 17DD-YF Vaccination. (**A**) Kinetics of serum biomarker signatures at distinct time points after primary 17DD-YF vaccination of Autoimmune Disease patients (AID, n = 140, D3–4 = light grey filled square, D5–6 = grey filled square, D7 = dark grey filled square and D14–28 = black filled circle) and Healthy Controls (HC, n = 21, D3–4 = light grey filled circle, D5–6 = grey filled circle, D7 = dark grey filled circle and D14–28 = black filled circle). The results are expressed as the proportion of subjects with increased biomarker levels (baseline fold change values > 1). Data analysis was carried out considering the 50th percentile as the reference to identify the set of biomarkers with high proportion of subjects with levels above the global median cut-off along the kinetic timeline. Those biomarkers with proportion of subjects with levels above the global median cut-off were highlighted with gray scale rectangles. (**B**) Heatmaps were constructed considering the baseline fold change values at each time point along the kinetic follow-up (D3–4, D5–6, D7 and D14–28). This approach was employed to draw the overall change in the serum biomarkers profile after primary 17DD-YF vaccination of Autoimmune Disease patients (AID, n = 140) and Healthy Controls (HC, n = 21). Data interpretation was carried out based on the color keys employed to underscore the baseline fold value = 1.0 as the reference for unaltered levels (black filled square), the baseline fold value < 1.0 for decreased levels (green filled square) and the baseline fold value > 1.0 for increased levels (red filled square), according to the paired sample collected at D0.

The serum biomarker signature of patients with autoimmune diseases were assessed at distinct time points after planned primary 17DD-YF vaccination and the results presented in Fig. 4. Data analysis demonstrated that, in general, AID patients exhibited a more prominent increased in serum biomarkers levels as compared to health controls. In fact, a massive up-regulation of several soluble mediators was observed in AID patients at Day3–4 and Day5–6 with subsequent decrease at Day7 towards Day14–28 (Fig. 4A). Noteworthy, was that while HC presented a synchronic increase of IFN-γ at the peak of viremia (Day5–6) with late increase of IL-10 at Day7, AID patients displayed a simultaneous and persistent increase of IFN-γ and IL-10 at the peak of viremia (Day5–6) up to Day7 after vaccination (Fig. 4A). Heatmap analysis further illustrated these synchronic/asynchronous phenomena observed for the IFN-γ and IL-10 rhythm nearby the viremia peak (Fig. 4B).

### Serum biomarker signatures after planned primary 17DD-YF vaccination according to the type of autoimmune disease

**Figure 5:**
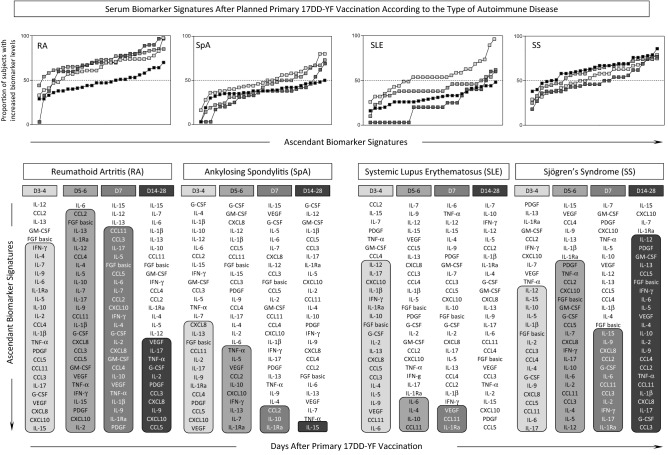
Serum Biomarker Signatures After Planned Primary 17DD-YF Vaccination According to the Type of Autoimmune Disease. Kinetics of serum biomarker signatures at distinct time points after primary 17DD-YF vaccination of Autoimmune Disease patients (D3–4 = light grey filled square, D5–6 = grey filled square, D7 = dark grey filled square and D14–28 = black filled square) categorized according to the type of Autoimmune Disease: Rheumatoid Arthritis (RA, n = 38), Spondyloarthritis (SpA, n = 51), Systemic Lupus Erythematosus (SLE, n = 21) and Sjögren’s Syndrome (SS, n = 30). The results are expressed as the proportion of subjects with increased biomarker levels (baseline fold change values > 1). Data analysis was carried out considering the 50th percentile as the reference to identify the set of biomarkers with high proportion of subjects with levels above the global median cut-off along the kinetic timeline. Those biomarkers with proportion of subjects with levels above the global median cut-off were highlighted with gray scale rectangles.

Serum biomarker signatures were further assembled for AID patients according to the type of autoimmune disease (Fig. 5). The results demonstrated that SpA and SLE patients presented early increase in serum biomarkers upon planned primary 17DD-YF vaccination, more prominent at Day3–4 with subsequent shift towards unaltered baseline fold changes in most soluble mediators at Day14–28 (Fig. 5). Conversely, RA and SS subgroups exhibited a robust and persistent increase in serum biomarkers upon planned primary 17DD-YF vaccination. While the RA subgroup still presented a decrease in several biomarkers later at Day14–28, the SS subgroup maintained most serum biomarkers elevated throughout the kinetic timeline after planned primary 17DD-YF vaccination (Fig. 5). Of note, was the finding that IFN-γ levels increase at the peak of viremia (Day5–6) in most AID subgroups, except for the SLE subgroup which presented IFN-γ up-regulation early at Day 3–4 (Fig. 5 and Supplementary Figure 1).

### Serum biomarker signatures in patients with autoimmune disease according to the neutralizing antibody status after planned primary 17DD-YF vaccination

**Figure 6:**
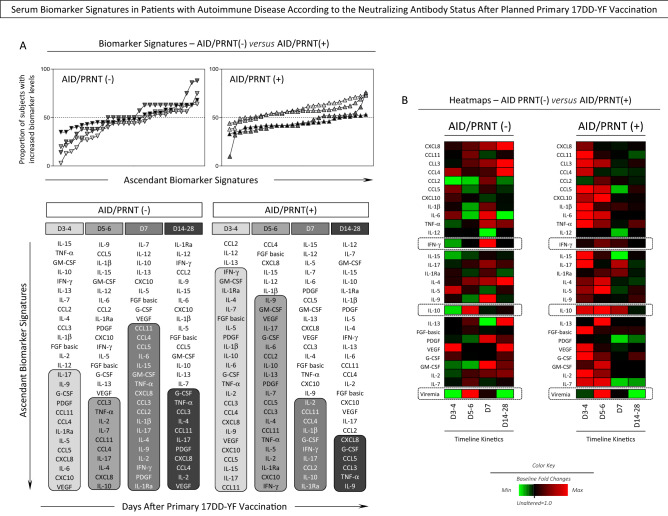
Serum Biomarker Signatures in Patients with Autoimmune Disease According to the Neutralizing Antibody Status After Planned Primary 17DD-YF Vaccination. (**A**) Kinetics of serum biomarker signatures at distinct time points after primary 17DD-YF vaccination of Autoimmune Disease patients categorized according to the PRNT status after primary 17DD-YF vaccination: AID/PRNT(−) (n = 32, D3–4 = light grey filled inverted triangle, D5–6 = grey filled inverted triangle, D7 = dark grey filled inverted triangle and D14–28 = black filled inverted triangle) and AID/PRNT(+) (n = 108, D3–4 = light grey filled triangle, D5–6 = grey filled triangle, D7 = dark grey filled triangle and D14–28 = black filled triangle). The results are expressed as the proportion of subjects with increased biomarker levels (baseline fold change values > 1). The results are expressed as the proportion of subjects with increased biomarker levels (baseline fold change values > 1). Data analysis was carried out considering the 50th percentile as the reference to identify the set of biomarkers with high proportion of subjects with levels above the global median cut-off along the kinetic timeline. Those biomarkers with proportion of subjects with levels above the global median cut-off were highlighted with gray scale rectangles. (**B**) Heatmaps were assembled for subgroups of Autoimmune Disease patients considering the PRNT status after vaccination [AID/PRNT(−), n = 32; AID/PRNT(+), n = 108] and according to the type of autoimmune disease [Rheumatoid Arthritis (RA, n = 38), Spondyloarthritis (SpA, n = 51), Systemic Lupus Erythematosus (SLE, n = 21) and Sjögren’s Syndrome (SS, n = 30)]. Data interpretation was carried out based on the color keys employed to underscore the baseline fold value = 1.0 as the reference for unaltered levels (black filled square), the baseline fold value < 1.0 for decreased levels (green filled square) and the baseline fold value > 1.0 for increased levels (red filled square), according to the paired sample collected at D0.

In order to further characterize the changes in serum biomarkers observed after planned primary 17DD-YF vaccination and its association with the post vaccination PRNT status, the AID patients were categorized as AID/PRNT(−) and AID/PRNT(+) and the fold changes in biomarker levels compared between AID subgroups (Fig. 6). Data analysis showed that AID/PRNT(+) exhibited an earlier serum biomarker response with higher fold change values at Day3–4 and Day5–6 with following decrease in a range of soluble mediators at Day7 towards Day14–28 (Fig. 6A). On the other hand, AID/PRNT(−) presented higher fold change in serum biomarkers at late time points with a progressive increase towards Day7, maintaining a sustained up-regulation of serum biomarkers up to Day14–28 (Fig. 6A). These findings suggest the occurrence of putative immunomodulatory effect of live attenuated 17DD-YF vaccine associated with the seroconversion status observed in AID (Fig. 6A).

Heatmap analysis further corroborates these findings according the color keys representing biomarkers with unaltered levels (black, baseline fold values = 1.0), decreased levels (green, baseline fold values < 1.0) and the increased levels (red, baseline fold values > 1.0) (Fig. 6B). AID/PRNT(−) exhibited an increase of IL-10 concomitant with the peak of viremia (Day5–6) and a late increase of IFN-γ at Day7. Conversely, AID/PRNT(+) patients displayed an early and persistent increase of IL-10 at Day3–4, Day5–6 and Day7 around the peak of viremia, maintaining the IFN-γ up-regulation concomitant with the peak of viremia (Day5–6) (Fig. 6B).

## Discussion

The 17DD-YF vaccine induces a safe and effective protective immunity in healthy vaccinees leading to high levels of protection in healthy adults (95–98%) resultant of robust humoral and cellular immunity^7,15–19^. Recent evidences have shown that planned primary 17DD-YF vaccination is safe and immunogenic but lower seropositivity rate is overall observed in AID patients upon planned 17DD-YF primary vaccination^14^.

The present study aimed to investigate whether the serum biomarkers of immune response orchestrate the distinct seroconversion rates in AID patients, leading to positive or negative results in plaque reduction neutralization test, depending on the type of disease. For this purposes, four subgroups of AID patients (RA, SpA, SLE and SS) were enrolled in an observational phase IV controlled prospective analysis to quantify the serum biomarker timeline profiles (D0, D3–4, D5–6, D7, D14–28) along with the 17DD-YF RNAnemia, taking into account the seropositivity rates of neutralizing antibodies (PRNT ≥ 1:50) after planned primary 17DD-YF vaccination.

Overall, our results demonstrated lower PRNT seropositivity rate in AID patients with decreased GeoMean titers as compared to HC. Campi-Azevedo et al.^20^ have previously shown that distinct patterns of viremia kinetics may affect the 17DD-YF vaccine immunogenicity in healthy adults. These authors postulated that early viremia peak after primary vaccination was associated with higher seroconversion rates, while lower and late viremia peak was observed in primary vaccinees exhibiting lower seroconversion rates. According to these authors a sub-optimal antigen exposure would lead to the generation of impaired protective humoral response, regardless the ability of live-attenuated virus to replicate and amplifies the antigenic exposure^20^. Our data demonstrated that the viremia levels at peak did not differ between AID/PRNT(−) and AID/PRNT(+) as compared to HC. Therefore, distinct viremia profiles seem to not substantiate the differences observed in the seroconversion rates in subgroups of AID.

It has been proposed, besides the viremia profile, the analysis of systemic immunological mediators should be also evaluated together with PRNT titers to better understand the immune response triggered by the 17DD-YF vaccine and its relationship with the development of protective antibodies^20^. A detailed analysis of several serum biomarkers, including chemokines and cytokines together with the viral load profile and the levels of neutralizing antibodies was carried out in healthy adults receiving distinct doses of 17DD-YF vaccine in a time and dose dependent fashion. Distinct kinetic profiles of chemokines, pro-inflammatory and modulatory cytokines were observed according to the seroconversion rates^20^.

Our data demonstrated that, in general, the 17DD-YF vaccine triggered a more prominent serum biomarker response in AID as compared to HC, throughout the timeline kinetics. Remarkably, AID/PRNT(−) exhibited higher levels of several biomarkers prior vaccination as compared to AID/PRNT(+). These differences in baseline profiles of serum biomarkers suggested that an inflammatory status prior vaccination may orchestrate the lack of antibody response. The analysis of changes in serum biomarker levels throughout the time line kinetics showed that while the AID/PRNT(+) exhibited an earlier (Day3–4/Day5–6) higher fold changes in serum biomarker, the AID/PRNT(−) displayed higher fold changes at late time points (Day7/Day14–D28). Of note, a synchronic pattern of high IFN-γ levels at the day of peak viremia (D5–6) was observed in HC and AID/PRNT(+) groups, whereas an asynchronous IFN-γ response was reported for AID/PRNT-later at Day7. Interestingly, AID/PRNT(+), biomarkers tend to deflate the biomarker response at post-vaccination timeline highlighting a putative immunomodulatory effect of live attenuated 17DD-YF vaccine. This effect was not observed in AID/PRNT(−). The synchronic/asynchronous rhythm of pro-inflammatory/modulatory serum biomarkers detected along the timeline kinetic, particularly nearby the peak of viremia (Day5–6), may be associated with distinct seroconversion profile observed in AID subgroups.

Campi-Azevedo et al.^20^ have postulated that asynchronous production of IL-10 opposite to the viremia peaks and pro-inflammatory cytokines (TNF-α and IFN-γ). The hypothesis was that the decreased levels of IL-10 at the peak of viremia support a pro-inflammatory microenvironment to produce neutralizing antibodies^20^. Corroborating this hypothesis, it has been previously demonstrated that the lack of seroconversion after YF-17DD primary vaccination was associated with lower levels of pro-inflammatory cells (TNF-α + neutrophils and monocytes) and correlated with enhanced levels of IL-10 produced by CD8^+^ T-cells^21^.

A remark of the present investigation was the opportunity to perform a parallel analysis of YF-specific neutralizing antibody and the quantification of serum biomarkers along the kinetics timeline after planned 17DD-YF primary vaccination, focusing on patients with distinct types of AID. Our finding demonstrated that amongst AID subgroups, SpA and SLE patients exhibited the lowest seropositivity rate. The analysis of viremia levels at peak did not show differences amongst AID subgroups (RA, SpA, SLE and SS) as compared to HC. Again, the viremia profiles did not support the differences observed in the seroconversion rates in the subgroups of AID. The results comprising the serum biomarker signatures after planned primary 17DD-YF vaccination according to the type of autoimmune disease demonstrated that SpA and SLE patients presented early increase in serum biomarkers (Day3–4) with subsequent decrease in most soluble mediators (Day14–28). RA and SS subgroups exhibited a robust and persistent increase in serum biomarkers upon planned primary 17DD-YF vaccination. Noteworthy was that most AID subgroups exhibited a synchronic IFN-γ levels increase at the peak of viremia (Day5–6), except the SLE subgroup which displayed early up-regulation of IFN-γ at Day 3–4. However, unlikely observed for HC, all AID subgroups displayed an up-regulation of IL-10 at the peak of viremia.

In SLE patients, it is possible that the asynchronous IFN-γ production and the up-regulation of IL-10 at peak of viremia may explain the lower seroconversion rates observed. Kyogoku et al.^22^ have performed a detailed analysis describing the differences in the IFN signature in SLE patients as compared to healthy donors. Transcriptional responses of peripheral blood monocyte subsets and CD4^+^ T-cells demonstrated that IFN-associated gene signature was distinct in monocytes and CD4^+^ T-cells and from SLE as compared to healthy controls. In fact, it was observed that healthy donors, even days after YF vaccine immunization displayed a virus-induced monocyte and CD4^+^ T-cell signatures comprising only approximately 36–47% and 10% of the probe-sets identified in SLE, respectively^22^. The analysis of IFN signatures showed qualitative and quantitative differences in SLE, characterized by a complex compiled gene patterns with increased expression levels. In this sense the assigned “autoimmune-specific” IFN signatures observed in SLE patients may explain the early IFN-γ production observed in our results upon 17DD-YF primary vaccination.

As far as the SpA patients, despite the synchronic IFN-γ levels increase at the peak of viremia (Day5–6), the concomitant up-regulation of IL-10 at Day5–6 may contribute to the lower seroconvertion rate observed. It is possible that a hyper-inflammatory state prior 17DD-YF vaccination plays a role tuning the immune response in these patients. Increasing evidences from mouse studies have been accruing to support that cytokine dysregulation, especially the TNF-α/IL-17/IL-23 axes is an emerging picture of aberrant immune hyper-activation in human SpA. As the role of inflammatory cytokines (TNF-α, IL-17 and IL-23) may differ across the group of spondyloarthritis diseases^23,24^, other factors may impact the seroconversion rates in SpA patients, such as: the disease activity at baseline as well as the type of immunomodulatory therapy schemes prior vaccination. The influence of such conditions remains to be elucidated in further investigations.

The present work has some limitations. The sample size, especially the number of AID/PRNT(−) subjects is relatively small requiring further validation in larger study investigations. Moreover, we did not analyze the cellular immunity profile in AID subgroups upon planned 17DD-YF primary vaccination. In this study, we were unable to categorize the AID patients according to the use of non-biological or biological immunomodulatory therapy, due to restrictions in sample size. Furthermore, we did not investigate the impact of disease activity on the effectiveness of planned 17DD-YF primary vaccination neither follow-up the long-term changes in disease activity over time after vaccination. These issues are currently under investigation in the ongoing multicenter study referred as OCAMO Project – Brazilian cohort of immunogenicity and safety of live-attenuated vaccines, carried out by the Collaborative Group for the study of MMR (Measles, Mumps and Rubella) and Yellow Fever Vaccines.

Altogether these data suggested that inflammatory status prior vaccination, low IFN-γ at viremia peak and the occurrence of asynchronous biomarker storm after 17DD-YF vaccination orchestrate the seroconversion status of patients with autoimmune diseases upon planned primary 17DD Yellow Fever vaccination. It is possible that other factors such as disease activity as well as the use of non-biological or biological immunomodulatory therapy schemes prior vaccination may also impact the seroconversion profiles of AID subgroups. Future studies including these topics will further clarify the distinct seroconversion rates observed amongst types of AID as compared to healthy controls.

## Study population, material and methods

### Study population

The present study was carried out as a prospective non-interventional observational investigation carried out between March, 2017 and July, 2017 at the Rheumatology Outpatient Clinic of the Hospital Universitário Cassiano Antônio de Moraes (HUCAM/EBSERH) of the Federal University of Espírito Santo (UFES) in Vitória, Espírito Santo, Brazil. A total of 140 patients with Autoimmune Disease patients (AID; both gender; age ranging from 18 to 88 years) were enrolled as a convenience sampling that include: 38 with Rheumatoid Arthritis (RA), 51 with Spondyloarthritis (SpA), 21 with Systemic Lupus Erythematosus (SLE) and 30 with Sjögren’s Syndrome (SS). A group of 21 Healthy Controls without diagnosis of autoimmune diseases (HC; both genders; age ranging from 24 to 76 years), comprising subjects who spontaneously searched the HUCAM/EBSERH for routine YF primary vaccination was also included for comparative analysis. Details about demographic features of the study population are provided in the Supplementary Table 1.

All participants had no previous history of YF vaccination and received the primary dose of 17DD-YF vaccine (Bio-Manguinhos-FIOCRUZ) during the 2017 Brazilian YF vaccination campaign, coordinated by the State Government. The study was submitted and approved by the ethical committee of HUCAM-EBSERH/UFES (C.A.A.E 65,910,317.0.0000.5071, Approval number #2.411.738/2017) and registered at the Brazilian Registry of Clinical Trials (UTN# U1111-1217-6672/https://ensaiosclinicos.gov.br/rg/RBR-3875dd). Informed consent was obtained from all participants. All methods were executed in accordance with guidelines and regulations of Helsinki Declaration, the Brazilian ethical standards of scientific and the good clinical practices.

The inclusion criteria comprised: individuals older than 18 years, with no previous records of YF vaccination. The following criteria were considered for the volunteers comprising the AID group: patients who fulfilled the international classification criteria for autoimmune diseases diagnosis, based on the American College of Rheumatology and/or European League Against Rheumatism guidelines^25–30^; patients in remission or with low disease activity advised by a rheumatologist to receive planned 17DD-YF primary vaccination upon withdrawal of immunomodulatory therapy with interruption interval as specified by Brazilian recommendations for YF vaccination of AID patients^13^. Details about immunomodulatory therapy prior withdrawal are provided in the Supplementary Table 1. The exclusion criteria comprised: previous history of YF vaccination; medical advice to not receive the YF vaccine; subjects who refuse to participate in the study; primary immunodeficiency or immunosuppression by other causes: HIV carriers with CD4 count lower than 200 cells/mm^3^ or lymphocyte counts lower than 500 cells/mm^3^, low IgM or IgG levels (according to patient’s information for previous medical records); history of organ transplantation; neoplasia; subjects who received another vaccine simultaneously or within a 30 days interval.

**Figure 7:**
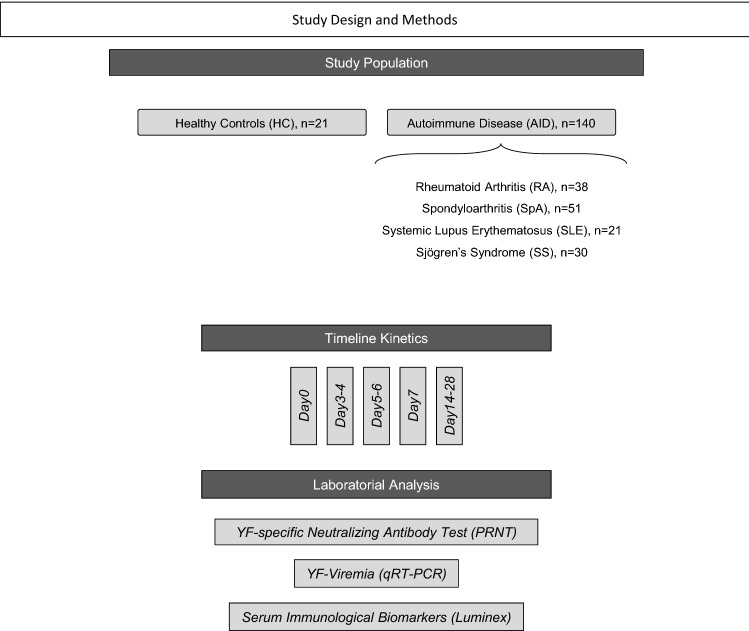
Study Design and Methods. This is a prospective non-interventional observational study carried out between March, 2017 and July, 2017 in Vitória, Espírito Santo, Brazil. A total of 140 patients with Autoimmune Disease patients (AID) including: Rheumatoid Arthritis (RA, n = 38), Spondyloarthritis (SpA, n = 51), Systemic Lupus Erythematosus (SLE, n = 21) and Sjögren’s Syndrome (SS, n = 30). A group Healthy Controls without diagnosis of autoimmune diseases (HC, n = 21) was also included. All participants received the primary dose of 17DD-YF vaccine (Bio-Manguinhos-FIOCRUZ) during the 2017 Brazilian YF vaccination campaign. Detailed inclusion and exclusion criteria are provided in Methods. Blood samples were collected from each participant at distinct time points, including: D0; Day3–4; Day5–6; Day7; Day14–D28. Serum samples were used for laboratorial analysis, including: YF-specific neutralizing antibodies analysis by Plaque Reduction Neutralizing Test (PRNT), YF viral RNAnemia (YF-Viremia) detection by quantitative real time PCR (qRT-PCR) and Serum Immunological Biomarkers quantification by Luminex Bio-plex assay.

The compendium of study design and methods is provided in the Fig. 7.

### Biological samples

Blood samples were collected from each participant at baseline (Day0) and at four consecutive scheduled time points, including: Day3–4; Day5–6; Day7; Day14–D28 after vaccination. Serum samples were used for laboratorial analysis as describe bellow, including: detection of YF-specific neutralizing antibodies by Plaque Reduction Neutralizing Test (PRNT), analysis of YF viral RNAnemia (YF-Viremia) by quantitative real time PCR (qRT-PCR) and quantification of Serum Immunological Biomarkers by Luminex Bio-plex assay. Serum samples were not collected from all participants at all time points due to ethical restriction or patients refuse.

### YF-plaque reduction neutralizing test (PRNT)

The quantification of YF-specific neutralizing antibody was carried out at the Laboratório de Tecnologia Virológica, Bio-Manguinhos/FIOCRUZ according to the standard protocol of plaque-reduction neutralization test (PRNT) described previously by Simões and colleagues, 2012^31^. The results were expressed as the reciprocal of the last serum dilution which reduced the plaque numbers in 50% (PRNT_50_) relative to the virus control included in each batch. Seropositivity was considered for PRNT titers higher than 1:50.

### YF-Viral RNAnemia quantifcation

The viremia level quantification (YF Viral RNAnemia) was performed by qRT-PCR assay as previously describe by Martins and colleagues, 2013^32^ using serum samples collected at four consecutive scheduled time points, including: Day3–4; Day5–6; Day7; Day14–D28 after vaccination. The assays were carried out at the Laboratório de Tecnologia Virológica, Bio-Manguinhos (LATEV, FIOCRUZ-RJ, Brazil) and the results were expressed as mean copies/mL at peak of viremia.

### Serum biomarkers measurements

The quantification of serum chemokines (CXCL8, CCL11, CCL3, CCL4, CCL2, CCL5, CXCL10), pro-inflammatory cytokines (IL-1β, IL-6, TNF-α, IL-12p70, IFN-γ, IL-15, IL-17), regulatory cytokines (IL-1Ra, IL-4, IL-5, IL-9, IL-10, IL-13) and growth factors (FGF-basic, PDGF, VEGF, G-CSF, GM-CSF, IL-2, IL-7) was performed at flow cytometry facility, Instituto René Rachou/FIOCRUZ-Minas, using the high-performance microbeads 27-plex array (Bio-Plex Pro Human Cytokine 27-plex Assay, Bio-Rad Laboratories, Hercules, CA, USA) according to the manufacture’s recommendations. The results are expressed in pg/mL according standard curve provided in the kit.

### Data analysis

#### Conventional statistical analysis

Data analysis were carried out using distinct approaches as follows: comparative analysis of the panoramic timeline kinetics of serum biomarkers for “AID *vs* HC” as well as for “AID/PRNT(−) *vs* AID/PRNT(+)” was performed considering the baseline fold value = 1.0 as the reference for decrease (< 1.0) or increase (> 1.0) in biomarker levels along the kinetic timeline. Comparisons of baseline levels of serum biomarkers from “AID/PRNT(−) *vs* AID/PRNT(+)” was assessed by Mann–Whitney test, considering statistical significance at *p* values < 0.05. The Shapiro–Wilk test was performed to define the normality of data distribution, which indicated non-parametric distribution. Multiple comparisons amongst groups were carried out (GraphPad Prism, version 5.03, San Diego, California, USA) by no-parametric Kruskal–Wallis test followed by Dunn’s post-test for sequential pairwise comparisons and a threshold *p* value of < 0.05 was considered for statistical significance.

#### Biomarkers signature and heatmap analysis

Biomarker signatures analyses were performed in Microsoft Excel 365 (Microsoft Corporation, Redmond, Washington, EUA) by first converting the baseline fold changes in biomarker levels along the kinetic timeline into categorical data using the baseline fold change values = 1.0 as the cut-off to calculate the proportion of subjects with increased biomarker levels. Heatmap profiles were assembled in Microsoft Excel 365(Microsoft Corporation, Redmond, Washington, EUA) using the baseline fold values obtained for each biomarker along the kinetic follow-up (D3–4, D5–6, D7 and D14–28), calculated according to the paired sample collected at D0. Baseline fold values = 1.0 were employed as the reference for unaltered levels while values < 1.0 and > 1.0 referred as decreased or increased levels, respectively.

## Supplementary Information

Supplementary Figure 1.

Supplementary Information 2.

## Data Availability

The datasets generated and/or analyzed during the current study are available from the corresponding author upon request.
