# Supplementary material for: Serum biomarker profile orchestrating the seroconversion status of patients with autoimmune diseases upon planned primary 17DD Yellow fever vaccination
Source: Sci Rep. 2021 May 17;11:10431. doi: 10.1038/s41598-021-89770-8 (PMC8128885; doi:10.1038/s41598-021-89770-8)
Supplement: Supplementary file 1 — Supplementary Figure 1. [file 41598_2021_89770_MOESM1_ESM.pdf]

Serum biomarker profile orchestrating the seroconversion status of patients with autoimmune diseases upon planned primary 17DD Yellow Fever vaccination

Ismael Artur da Costa-Rocha[a*], Ketty Lysie Libardi Lira Machado[b*], Ana Carolina Campi-Azevedo[a], Andréa Teixeira-Carvalho[a], Vanessa Peruhype-Magalhães[a], Sheila Maria Barbosa de Lima[c], Emily Hime Miranda[c], Gisela Freitas Trindade[c], Thays Zanon Casagrande[b], Samira Tatiyama Miyamoto[b], Sávio Carvalho Deotti[b], Rafaela Villa Real Barbosa[b], Priscila Costa Martins Rocha[b], Erica Vieira Serrano[b], Valquiria Garcia Dinis[b,d], Sônia Alves Gouvêa[b], Maria Bernadete Renoldi de Oliveira Gavi[b], Lidia Balarini da Silva[b], Ruben Horst Duque[b], Ana Paula Espíndula Gianordoli[b], Maria de Fatima Bissoli[b], Maria da Penha Gomes Gouvea[b], Lauro Ferreira da Silva Pinto-Neto[d], Ana Paula Neves Burian[e], Francieli Fontana Sutile Tardetti Fantinato[f], Gecilmara Salviato Pileggi[g], Licia Maria Henrique da Mota[h], Valéria Valim[b#] and Olindo Assis Martins-Filho[a#]

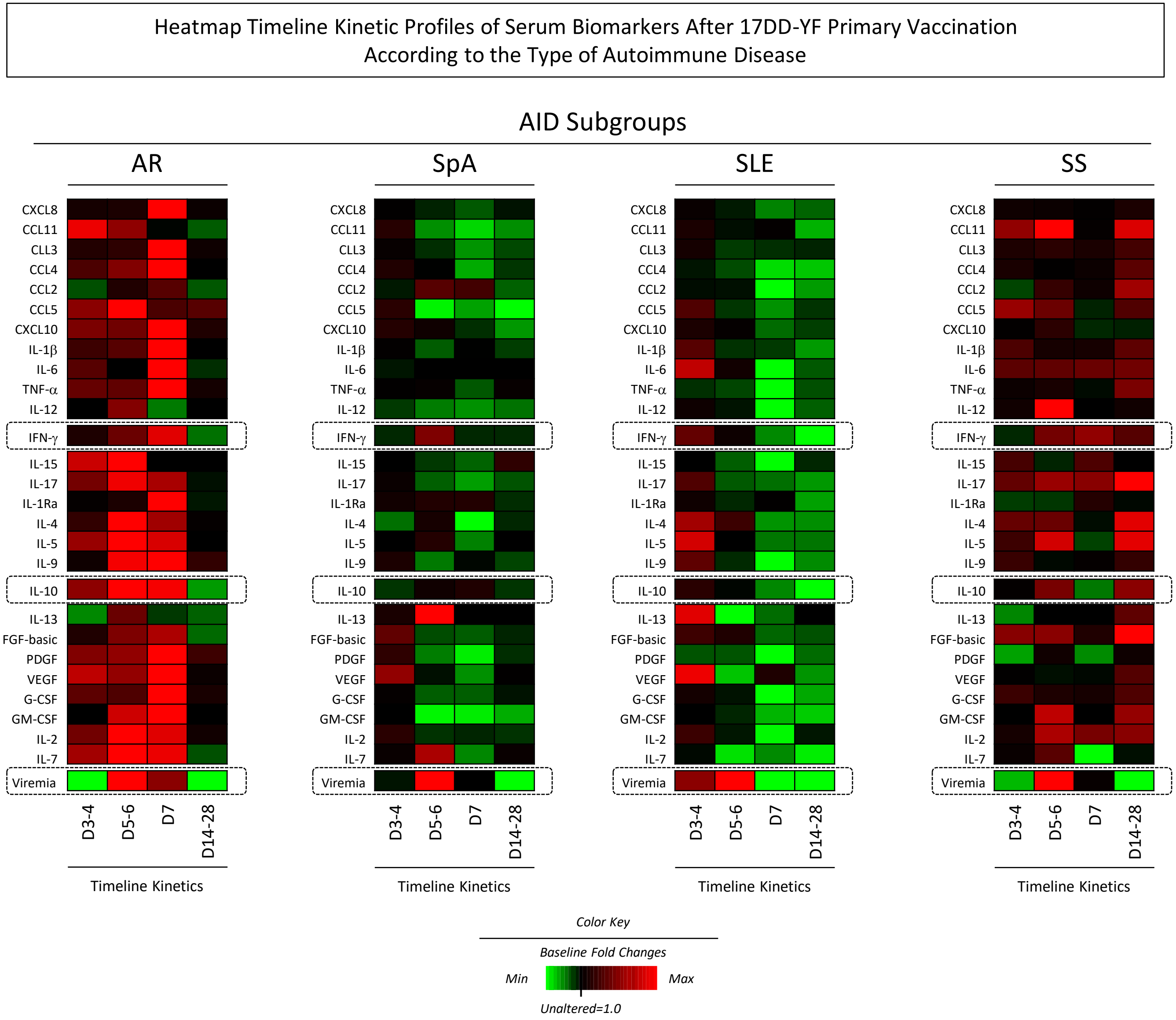

Supplementary Figure 1 - Heatmap Timeline Kinetic Profiles of Serum Biomarkers After 17DD-YF Primary Vaccination According to the Type of Autoimmune Disease. Heatmaps were constructed considering the baseline fold change values at each time point along the kinetic follow-up (D3-4, D5-6, D7 and D14-28). This approach was employed to draw the overall change in the serum biomarkers profile after primary 17DD-YF vaccination of Autoimmune Disease patients segregated according to the type of autoimmune disease [Rheumatoid Arthritis (RA, n=38), Spondyloarthritis (SpA, n=51), Systemic Lupus Erythematosus (SLE, n=21) and Sjögren's Syndrome (SS, n=30)]. Data interpretation was carried out based on the color keys were employed underscore the baseline fold value = 1.0 as the reference for unaltered levels (▬), the baseline fold value <1.0 for decreased levels (▬) and the baseline fold value >1.0 for increased levels (▬), according to the paired sample collected at D0.
