# Supplementary material for: Serum biomarker profile orchestrating the seroconversion status of patients with autoimmune diseases upon planned primary 17DD Yellow fever vaccination
Source: Sci Rep. 2021 May 17;11:10431. doi: 10.1038/s41598-021-89770-8 (PMC8128885; doi:10.1038/s41598-021-89770-8)
Supplement: Supplementary file 2 — Supplementary Information 2. [file 41598_2021_89770_MOESM2_ESM.pdf]

Serum biomarker profile orchestrating the seroconversion status of patients with autoimmune diseases upon planned primary 17DD Yellow Fever vaccination

Ismael Artur da Costa-Rocha[a*], Ketty Lysie Libardi Lira Machado[b*], Ana Carolina Campi-Azevedo[a], Andréa Teixeira-Carvalho[a], Vanessa Peruhype-Magalhães[a], Sheila Maria Barbosa de Lima[c], Emily Hime Miranda[c], Gisela Freitas Trindade[c], Thays Zanon Casagrande[b], Samira Tatiyama Miyamoto[b], Sávio Carvalho Deotti[b], Rafaela Villa Real Barbosa[b], Priscila Costa Martins Rocha[b], Erica Vieira Serrano[b], Valquiria Garcia Dinis[b,d], Sônia Alves Gouvêa[b], Maria Bernadete Renoldi de Oliveira Gavi[b], Lidia Balarini da Silva[b], Ruben Horst Duque[b], Ana Paula Espíndula Gianordoli[b], Maria de Fatima Bissoli[b], Maria da Penha Gomes Gouvea[b], Lauro Ferreira da Silva Pinto-Neto[d], Ana Paula Neves Burian[e], Francieli Fontana Sutile Tardetti Fantinato[f], Gecilmara Salviato Pileggi[g], Licia Maria Henrique da Mota[h], Valéria Valim[b#] and Olindo Assis Martins-Filho[a#]

Supplementary Table 1. Demographic and immunomodulatory therapy

Features	HC (n = 21)	AID (n = 140)	RA (n = 38)	SpA (n = 51)	SLE (n = 21)	SS (n = 30)
**Women/Man, Ratio (%)**	14/07 (67)	106/34 (76)	30/08 (79)	27/24 (53)	21/00 (100)	28/02 (93)
**Age, mean (SD), years**	54 (15)	49 (14)	52 (15)	48 (11)	44 (16)	53 (13)
**PRED, %**	-	7.9	10.5	2.0	28.6	0.0
**MTX, %**	-	35.0	57.9	33.3	14.3	23.3
**LFN, %**	-	10.0	21.1	7.8	4.8	3.3
**HCQ, %**	-	26.4	18.4	0.0	76.2	46.7
**SSA, %**	-	7.9	0.0	19.6	0.0	3.3
**NSAI,%**	-	10.7	10.5	17.6	9.5	0.0
**AZA, %**	-	2.9	0.0	0.0	19.0	0.0
**Biological Therapy[a], %**	-	22.9	26.3	43.1	0.0	0.0

Abbreviations: HC, Healthy Controls; AID, Autoimmune Disease Patients; RA, Rheumatic Arthritis; SpA, Spondyloarthritis; SLE, Systemic Lupus Erythematosus; SS, primary Sjögren's Syndrome; PRED, Prednisone; MTX, Methotrexate; LFN, Leflunomide; HCQ, Hydroxychloroquine; SSA, Sulfasalazine; NSAI, non-steroidal antinflammatory; AZA, Azathioprine. [a] Biological Therapy included: adalimumab, etanercept, infliximab, abatacept and rituximab.
